# Association between Left Atrial Deformation and Brain Involvement in Patients with Anderson-Fabry Disease at Diagnosis

**DOI:** 10.3390/jcm9092741

**Published:** 2020-08-25

**Authors:** Roberta Esposito, Camilla Russo, Ciro Santoro, Sirio Cocozza, Eleonora Riccio, Regina Sorrentino, Giuseppe Pontillo, Federica Luciano, Massimo Imbriaco, Arturo Brunetti, Antonio Pisani

**Affiliations:** 1 Departement of Clinical Medicine and Surgery, Federico II University Hospital, 80131 Naples, Italy; roberta.esposito1@unina.it; 2 Mediterranea Cardiocentro, 80122 Naples, Italy; 3 Department of Advanced Biomedical Sciences, Federico II University Hospital, 80131 Naples, Italy; camilla_russo@hotmail.it (C.R.); siriococozza@hotmail.it (S.C.); rejinasorrentino@gmail.com (R.S.); gpontillo@hotmail.it (G.P.); federicaa.luciano@gmail.com (F.L.); mimbriaco@hotmail.com (M.I.); brunetti@unina.it (A.B.); 4 Department of Public Health, Nephrology Unit, Federico II University Hospital, 80131 Naples, Italy; elyriccio@libero.it (E.R.); antonio.pisani13@gmail.com (A.P.)

**Keywords:** Anderson Fabry disease, echocardiography, left atrial strain, white matter lesions, fazekas’ score

## Abstract

Background: Anderson-Fabry disease (AFD) can induce both central nervous system white matter lesions (WMLs) and cardiac abnormalities including left atrial (LA) dysfunction. We sought to evaluate the possible interrelations of LA structure and function impairment with the presence of WMLs in AFD patients. Methods 22 AFD patients and 22 controls, matched for age and sex, underwent an echo-Doppler exam including quantification of peak atrial longitudinal strain (PALS). AFD patients underwent also a 3-T brain magnetic resonance imaging with a visual quantification of WMLs by Fazekas’ score (FS) on 3D FLAIR images. Results AFD patients had significantly higher left ventricular (LV) mass index (LVMi) and relative wall thickness, and lower PALS compared to controls. Among AFD patients, 9 showed a FS = 0, and 13 a FS > 1. AFD patients with FS ≥ 1 showed lower PALS (29.4 ± 6.7 vs. 37.2 ± 3.9%, *p* = 0.003) than those with FS = 0, without difference in LA volume index and LVMi. In AFD patients, FS was inversely related to PALS (r = −0.49, *p* < 0.0001), even after adjusting for LVMi (r = −0.43, *p* < 0.05). Conclusions In the absence of significant alterations in LA size, AFD patients had lower PALS compared to controls. The inverse association between PALS and presence of WMLs indicates a possible parallel early involvement of heart and brain.

## 1. Introduction

Anderson-Fabry disease (AFD) is a rare X-linked lysosomal storage disorder leading to severe multiorgan dysfunction and inducing premature death. The disease is due to mutation in a gene that encodes alpha-galactosidase A (α-GalA), a lysosomal enzyme [1], with subsequent accumulation of sphingolipids in the lysosomes of various tissues. In cardiomyocytes, the accumulation of sphingolipids causes inflammation, hypertrophy and interstitial fibrosis [2], occurring at the early disease stages, even in the absence of overt symptoms of organ failure. Patients frequently develop cardiac involvement, such as rhythm and conduction disturbances, progressive hypertrophic cardiomyopathy and both atrial and ventricular arrhythmias [3,4,5]. Since atrial fibrillation (AF) is a possible complication of AFD, possibly causing cerebral ischemic events, the assessment of left atrial (LA) structure and function should be part of the echocardiographic work-up in this clinical setting. Moreover, although transient brain ischemia and major stroke are the most paradigmatic sequelae of central nervous system involvement in AFD, non-specific periventricular and deep white matter lesions (WMLs) along with silent lacunar infarctions are far more common, arising also in asymptomatic or poorly symptomatic patients [6]. As in several other conditions, the presence of white matter hyperintensities, ischemic lacunae and/or prominent perivascular spaces are all suggestive of chronic small vessels disease. Limited subcortical infarcts might evolve both into lacunar cavities and white matter hyperintensity without apparent cavitation on T2 weighted (T2w) images [7]. At present time, the etiopathogenesis of these phenomena is still largely unclear, being only partially explained by Gb-3 accumulation within the endothelium [8]. Among different methods, assessing the extent of WMLs, Fazekas’ Score (FS), first proposed in 1987 [9], is one of the most used visual semi-quantitative scale to assess WML load by magnetic resonance imaging (MRI). This scale distinguishes deep and periventricular white matter, assigning to each a grading depending on the size and confluence of the WMLs [10,11]; in particular, the score is the sum of two 4-point scales (ranging from 0 to 3 each) assessing periventricular and deep white matter hyperintensities, with the total rate ranging from 0 to 6 (with higher values associated to higher lesion burden).

Little is known about the interaction between LA features and brain involvement in AFD patients. To date, the assessment of LA size and function can be performed by combining standard echo measurements with speckle tracking derived peak atrial longitudinal strain (PALS), an accurate and reproducible indicator of LA deformation [12]. Worthy of note, reduced PALS has been demonstrated to be associated with AF recurrence in the general population, after ablation or cardioversion [13,14]. PALS has been also evaluated in AFD, which was reduced in comparison with healthy controls [15]. The present study aimed to evaluate possible association between LA structure and function and presence of WML assessed by FS in patients suffering from AFD.

## 2. Materials and Methods

### 2.1. Study Population

We enrolled 22 newly diagnosed and enzyme replacement therapy-naive patients affected by genetically confirmed AFD classical variant. The study was approved by the local Ethical Committee (protocol number 1233/19). We excluded patients with relevant cardiac or central nervous system signs/symptoms, patients affected by cardiac risk factors (i.e., diabetes mellitus, dyslipidemia, hypertension), patents already under enzyme replacement therapy, patients with evidence of stroke at brain MRI, and patients with poor echocardiographic imaging. All patients had confirmed diagnosis of AFD: for all subjects the evaluation of α-GalA activity and the galactosidase alpha (*GLA)* gene test was performed using the Dried Blood Filter Paper test. In male patients, diagnosis was made by demonstration of a deficient activity of alfa-galactosidase in plasma; female patients were directly tested for possible mutations in *GLA* gene, and then evaluated for α-GalA activity when positive at genetic test. All AFD patients underwent a comprehensive evaluation of AFD target organs. This included clinical examination, 12 leads resting electrocardiogram (ECG) and blood draw for routine biochemical determinations. Serum LysoGb3 levels were measured by highly sensitive electrospray ionization liquid chromatography tandem mass spectrometry [16] in a single laboratory (Centogene, Rostock, Germany, reference normal values ≤ 1.8 ng/mL). Other laboratory values were measured using standard laboratory techniques of Federico II University hospital. Renal function was expressed as estimated glomerular filtration rate, calculated with the Chronic Kidney Disease Epidemiology Collaboration equation. The control group consisted of 22 age and gender-matched healthy volunteers. Clinical examination, 12-lead resting ECG, standard and advanced echocardiographic exam and brain MRI were acquired for each patient. All participants gave written informed consent to the procedures before entering the study.

### 2.2. Echocardiography

Echo Doppler exams were performed by a Vivid E95 ultrasound machine (Horten, Norway) equipped with a 2.5 MHz phased-array transducer according to the American Society of Echocardiography (ASE)/European association of Cardiovascular Imaging (EACVI) standardization [17]. Blood pressure (BP) and heart rate were measured at the beginning of each echocardiographic exam. The quantitative analysis of the left ventricle, including left ventricular (LV) mass, relative wall thickness and 2D derived ejection fraction, was performed in agreement of 2015 ASE/EACVI recommendations on chamber quantification [18]. LV mass was normalized for height powered to 2.7 and LV hypertrophy defined as LV mass index (LVMi) >47 g/m^2.7^ in women and >50 g/m^2.7^ in men [19]. LA volume was calculated by using area-length method (average of 4- and 2-chamber view) and indexed for body surface area [18]. Transmitral E/A ratio, E velocity deceleration time (DT), pulsed tissue Doppler of septal and lateral annulus (early diastolic velocity [e’]), average E/e’ ratio and tricuspid regurgitation (TR) jet peak velocity were determined in apical 4-chamber view as recommended [20]. The determination of pulmonary arterial systolic pressure was based on the TR peak velocity and adding to that right atrial pressure to the estimated TR retrograde gradient. Right atrial pressure was estimated according to ASE guidelines, based on the size of the inferior vena cava and the degree of its inspiratory collapse during normal respirations [21]. All the echo Doppler measurements were averaged from three cardiac cycles.

**Figure 1:**
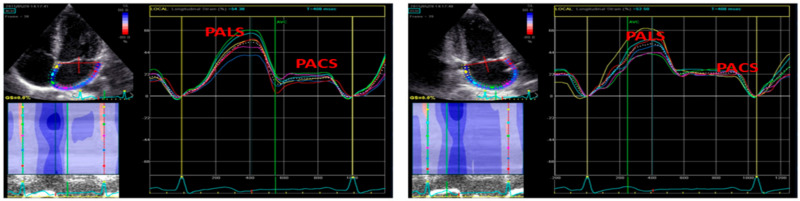
Speckle tracking echocardiography (STE) derived methodology to calculate left atrial (LA) strain in apical 4- and 2-chamber views. Left panel—upper left corner: two-dimensional echocardiography apical 4-chamber view, bottom left corner: color rendering of LA strain variation during cardiac cycle, right side: LA strain curves with peak atrial longitudinal strain (PALS) and peak atrial contraction strain (PACS). Right panel—upper left corner: two-dimensional echocardiography apical 2-chamber view, bottom left corner: color rendering of LA strain variation during cardiac cycle, right side: LA strain curves with peak atrial longitudinal strain (PALS) and peak atrial contraction strain (PACS).

Speckle-tracking echocardiography acquisition and post-processing (EchoPac, GE, Milwaukee, WI, USA) were performed in apical long-axis, 4-chamber, and 2-chamber views of the conventional 2D gray scale images with a stable ECG recording, according to standardized procedures of our laboratory [22,23]. Global Longitudinal strain (GLS) was calculated by averaging all values of regional peak systolic by a semi-automatic 2D strain software [24]. LA strain was evaluated by 2D speckle tracking recording in the apical 4- and 2- chamber views [25]. LA endocardial border was manually traced, delineating a region of interest (ROI) which consists of 6 segments. After tracking, quality analysis was done by manual adjustment of the ROI, and the longitudinal strain curves generated by the software. Peak atrial longitudinal strain (PALS), measured at the end of the reservoir phase, and peak atrial contraction strain (PACS), obtained during LA systole, were determined as the average of regional LA measurements taken in apical 4- and 2-chamber views [24,25,26] (Figure 1).

### 2.3. Brain MRI Data Acquisition and Interpretation

All AFD patients underwent brain MRI examination on the same 3T scanner (Trio, Siemens Medical Systems, Erlangen, Germany). The scanning protocol included a volumetric Fluid Attenuated Inversion Recovery (FLAIR) sequence to determine high signal intensity regions within normal white matter, along with an axial Diffusion Weighted Imaging (DWI) sequence to differentiate chronic hyperintense lesions from acute ischemic events. FS, used as indicator of small vessel impairment of central nervous system, was calculated by the agreement of two experienced neuroradiologists on the 3D FLAIR images according to the following scheme:

− deep white matter:
  (1) no WMLs;
  (2) punctate foci of hyperintensity;
  (3) beginning confluent WMLs;
  (4) large confluent WMLs;
− periventricular white matter:
  (1) no WMLs;
  (2) periventricular caps;
  (3) smooth periventricular “halo”;
  (4) irregular periventricular WMLs extending into deep white matter.

**Figure 2:**
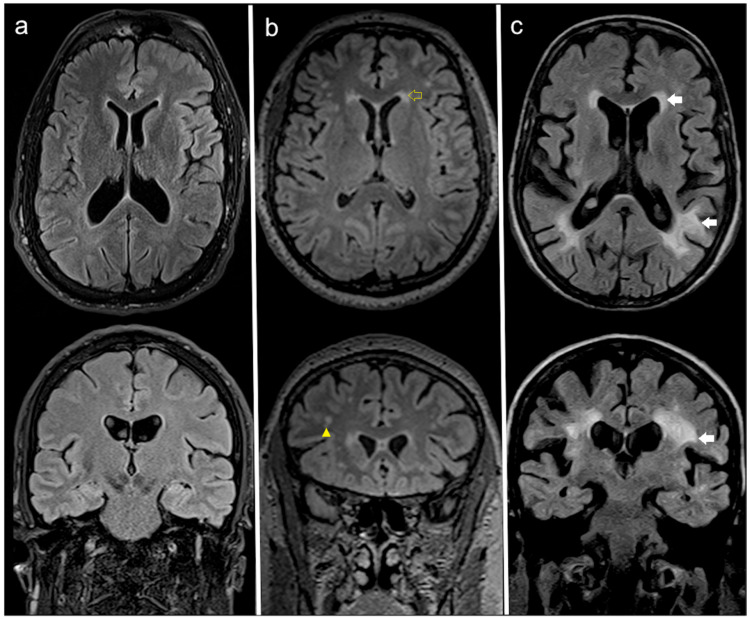
Axial (top) and coronal (bottom) FLAIR MRI at the most representative levels of three AFD patients with different degrees of white matter (WM) damage, respectively: (**a**) no WM involvement (52 years old woman with FS = 0); (**b**) moderate WML load (44 years old male patients scoring a FS = 3, with punctate foci of hyperintensity affecting the deep WM—yellow arrowhead and smooth bilateral periventricular “halo”—yellow empty arrow); (**c**) high WML load (59 years old man scoring FS = 6, with largely confluent asymmetrical WMLs irregularly extending from periventricular to deep white matter white arrows and some scattered lacunar infarcts not shown). Legend: FLAIR = fluid attenuated inversion recovery; MRI = magnetic resonance imaging; AFD = Anderson-Fabry disease; WML = white matter lesion; FS = Fazekas’ score.

Small silent ischemic lacunae, when present, were considered as part of the white matter hyperintensity. The overall score was then calculated as the sum of the deep white matter and the periventricular white matter values [9,27]. Figure 2 shows AFD patients with different degrees of white matter damage.

### 2.4. Statistical Analysis

Statistical analysis was performed by SPSS package, release 12 (SPSS Inc, Chicago, Illinois, USA). Data are presented as mean value ± SD. Descriptive statistics were obtained by one factor ANOVA and X2-distribution with computation of exact *p* value by Monte Carlo method. Univariate correlates of a given variable were evaluated by least squares linear regression. The null hypothesis was rejected at 2-tailed *p* < 0.05.

## 3. Results

### 3.1. Standard and Speckle Tracking Echocardiography

The characteristics of the study population are reported in Table 1. The two groups were comparable for heart rate and BP, whereas body mass index was marginally higher in AFD patients (*p* < 0.05).

Table 2. shows standard echo-Doppler and STE analysis. As expected, LVMi and relative wall thickness were higher in AFD patients than in controls (*p* < 0.002 and *p* < 0.001 respectively). Six AFD patients (27%) presented clear-cut LV hypertrophy. Also GLS was reduced in AFD patients (*p* < 0.01). The difference of LA volume index between the two groups did not achieve the statistical difference. PALS, but not PACS, was lower in AFD patients than in controls (*p* < 0.0001).

### 3.2. Brain MRI

Among the 22 AFD patients, WMLs were distributed as follows: 9 patients showed a FS = 0, 9 a FS = 1, 1 a FS = 2, 1 a FS = 3, 1 a FS = 5 and 1 a FS = 6, whereas none had a FS = 4. Small silent ischemic lesions were evident only in 3 AFD patients, one with FS = 0, one with FS = 5 and one with FS = 6. No sign of acute ischemic event was detected on DWI.

**Figure 3:**
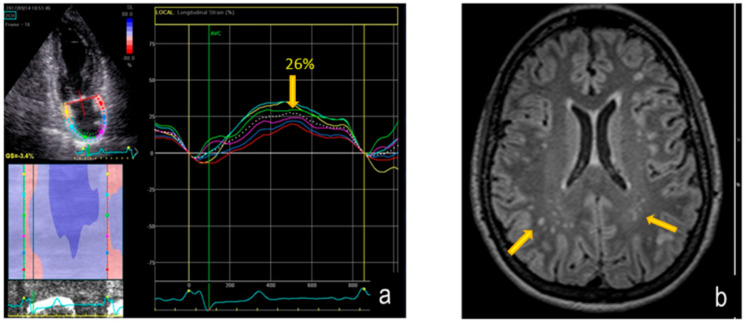
Reduction of PALS: upper left corner: two-dimensional echocardiography apical 2-chamber view, bottom left corner: color rendering of LA strain variation during cardiac cycle, right side: LA strain curves (**a**) and parallel axial FLAIR MRI image (**b**) showing multiple white matter hyper intensities lesions (arrows) in AFD patient. Fazekas score is = 1. Legend: PALS = peak atrial longitudinal strain; LA= left atrium; FLAIR = fluid attenuated inversion recovery; MRI = magnetic resonance imaging; AFD = Anderson-Fabry disease.

An example of AFD patient with white matter involvement on axial FLAIR MRI and reduced PALS is shown in Figure 3. By dividing AFD patients according to FS (<1 normal, ≥1 abnormal), the 13 patients with FS ≥ 1 showed a lower PALS (*p* = 0.0003) in comparison with FS < 1, without significant difference of LA volume index (Table 3). The other echocardiographic measurements, including LVMi and GLS, did not differ significantly between the two subgroups.

**Figure 4:**
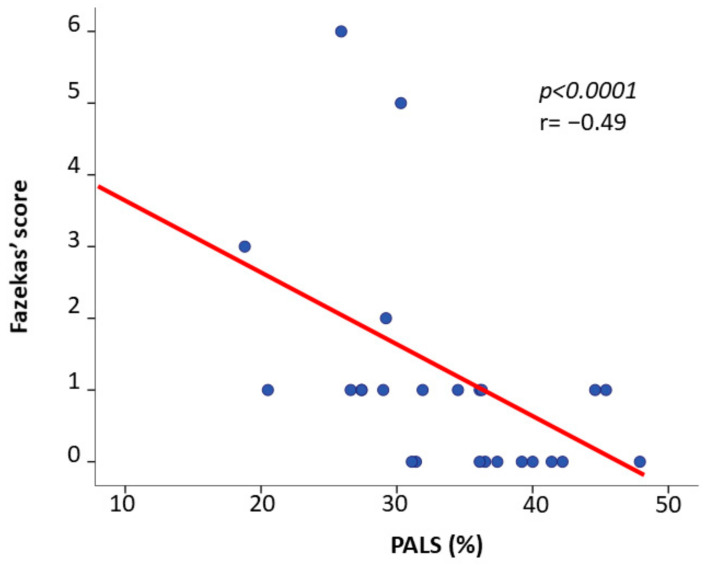
Scatterplot and regression line of the inverse relation between peak atrial longitudinal strainand Fazekas Score in Anderson-Fabry Disease patients.

In the pooled AFD population FS was positively related with PALS (r = −0.49, *p* < 0.0001) (Figure 4). This relation remained significant even after adjusting for LVMi (r = −0.43, *p* < 0.05). FS was not significantly related with LVMi (r = 0.29, *p* = 0.148), relative wall thickness (r = 0.25, *p* = 0.23), ejection fraction (r = 0.10, *p* = 0.66), transmitral E/A ratio (r = 0.11, *p* = 0.62), E/e’ ratio (r = 0.23, *p* = 0.28), LAVi (r = 0.10, *p* = 0.68), and GLS (r = 0.10, *p* = 0.63). No correlation was found between FS and LysoGB3 and between PALS and LysoGB3.

## 4. Discussion

To the best of our knowledge, this is the first study to explore the relationships between LA function deterioration and brain involvement, i.e., presence and extension of WMLs evaluated through the FS, in AFD treatment-naïve patients. The present study demonstrates that (I) treatment-naive AFD patients at diagnosis show a significant reduction of GLS and PALS in comparison with healthy controls, whereas ejection fraction, PACS and LA volume index do not differ between the two groups; (II) patients with greater PALS impairment present a larger involvement of the central nervous system according to FS (≥1); (III) an inverse relation between PALS and FS is detectable in AFD patients.

Speckle tracking-derived LA strain is a relatively new parameter of LA function, whose reference normal values have been recently defined [28]. The ability of PALS in the early detection of LA function impairment is already known, it preceding changes in traditional two-dimensional measures of LA size [29]. Previous studies showed changes of LA structure and function, including a reduction of PALS in patients with AFD compared to healthy controls [15,30,31]. In the present study AFD patients did not exhibit significant differences in LA size, in contrast with a previous work showing a substantial LA dilation even in AFD without LV hypertrophy [30]. However, the evidence of LA dilation in AFD remains controversial, as in a cardiac MRI study LA volumes of the AFD group were similar to those seen in the healthy control group [32]. In our AFD patients, PALS, but not PACS, was substantially lower than in healthy controls. PALS and PACS have different physiopathologic meanings, since PALS represents LA reservoir function, depending also on LA compliance, whereas PACS corresponds to LA systole. In the general population, reduced LA compliance, as assessed by PALS impairment, is related to the extent of LA remodelling [14,33]. It is conceivable that the early impairment of LA reservoir function and compliance in AFD could be attributable to the accumulation of glycosphingolipids in LA tissue, which was already observed by myocardial biopsy and may be also responsible of lone atrial fibrillation onset [34].

MRI derived WMLs of the brain, often unexpectedly observed even in the normal population, can be evaluated by visual semi-quantitative scoring such as the FS [9,10,11]. This can be applied to images of variables quality and different scanners, with the advantage of including not only the abnormality extension but also the lesion location and texture. In AFD patients, the brain alteration, typically sparing midline or infra-tentorial structures [35], is mainly due to cerebral micro-vessel involvement and endothelial dysfunction own of the disease [36,37,38]. Brain alteration is also associated both to a progressive subtle glycosphingolipid accumulation in blood vessels wall and to the possible ischemic areas due to thromboembolic events in patients experiencing AF [39]. Notably, AFD-induced brain abnormalities have been found to be associated with an increased risk of stroke in absence of focal neurological symptoms [40]. In the present study, a variable degree of FS was observed in AFD patients since 9 showed a FS = 0 and 13 had FS ≥ 1, whereas no sign of acute ischemic event was detected on DWI.

It is worthy of note that PALS was the only echocardiographic variable able to differentiate AFD patients according to FS, it being substantially reduced in those with higher score. PALS reduction was evident in AFD patients who also had greater progression of brain disease in terms of WMLs as evidenced by FS. In addition, a significant inverse correlation between PALS and FS was observed: the greater PALS impairment the higher FS. No other echo parameter was related to FS. Although it is impossible to establish a certain cause-effect mechanism between these two findings, this association can at least be partially explained by a simultaneous accumulation of glycosphingolipids in both LA and brain districts, as part of multi-organ involvement own of the disease.

### Study Limitations

Although easy to assess, FS is not completely reliable in relation with its subjective interpretation and consequent suboptimal intra-observer reproducibility [11]. Moreover, although chronic small vessel ischemia represents the most common cause, FS is unable to distinguish from other underlying mechanisms (demyelination and gliosis) possibly inducing WMLs. It also failed to distinguish AFD patients from non-AFD acute cerebrovascular events patients [27]. However, in the present study diagnosis of AFD was done by demonstration of a deficient activity of alfa-galactosidase in plasma for men, and by genetic analysis in women.

Small number of study population could be considered another limitation, however it is necessary to take into account that AFD is a rare disease.

Another limitation of the study is the significant difference in BMI between study groups that were age- and gender-matched, although this limitation did not impact on echocardiographic parameters that were all indexed by body surface area.

## 5. Conclusions

Our results confirm the concept that PALS may be considered an early predictor of target organ damage in AFD patients [15,30,31]. On the grounds of the significant association found in the present study between PALS reduction and FS scoring, it is conceivable that AFD patients with PALS abnormalities could be addressed to the performance of brain MRI in order to detect parallel involvement of white matter and initiate a very early enzyme replacement therapy, in agreement with the European Fabry Working Group recommendations [41,42]. This kind of therapy has in fact demonstrated to exert a protective effect on central nervous system, by reducing the stroke risk and stabilizing WMLs progression [43] and could be therefore promoted in AFD patients with LA strain dysfunction.

## Figures and Tables

**Table 1:** Characteristics of the study population.

Characteristics	AFD (*n* = 22)	Controls (*n* = 22)	*p*-Value
Gender (M/F)	14/8	14/8	1.00
Age (years)	39.0 ± 14.6	39.1 ± 14.4	0.99
BMI (Kg/m^2^)	26.1 ± 4.3	23.6 ± 2.5	<0.05
Systolic BP (mmHg)	124.7 ± 17.8	118.4 ± 8.8	0.14
Diastolic BP (mmHg)	76.7 ± 10.8	74.3 ± 6.9	0.37
HR (bpm)	71.4 ± 9.8	73.2 ± 7.6	0.56

AFD = Anderson-Fabry disease, BMI = body mass index, BP = Blood pressure, HR = heart rate.

**Table 2:** Echo-Doppler data of the study population.

Characteristics	AFD (*n* = 22)	Controls (*n* = 22)	*p* Value
LVMi(g/m^2.7^)	37.0 ± 12.5	27.0 ± 4.6	<0.002
RWT	0.34 ± 0.6	0.29 ± 0.3	<0.001
LV EF (%)	64.1 ± 3.8	62.6 ± 3.8	0.19
GLS (%)	21.9 ± 1.9	23.3 ± 1.7	<0.01
Transmitral E/A ratio	1.4 ± 0.44	1.3 ± 0.32	0.21
E velocity DT (msec)	202.5 ± 44.5	202.1 ± 37.1	0.96
E’ velocity (cm/sec)	0.78 ± 0.21	0.80 ± 0.21	0.64
E/e’ ratio	6.4 ± 1.2	5.6 ± 1.1	0.22
PASP (mmHg)	28.4 ± 6.2	26.2± 4.5	0.20
LAVi (ml/m^2^)	29.3 ± 8.6	25.3 ± 5.3	0.07
PALS	32.6 ± 6.9	42.2 ± 6.6	<0.0001
PACS	13.7 ± 3.7	16.3 ± 4.0	0.25

AFD = Anderson-Fabry disease, LVMi = left ventricular mass index, RWT = relative wall thickness, LV EF = Left ventricular ejection fraction, GLS = Global longitudinal strain, DT= deceleration time, PASP = pulmonary artery systolic pressure, LAVi = Left atrial volume index, PALS = Peak atrial longitudinal strain, PACS = Peak atrial contraction strain.

**Table 3:** Echocardiographic measurements according to FS in AFD patients.

Variables	FS ≥ 1 (*n* = 13)	FS < 1 (*n* = 9)	*p*-Value
Age (years)	43.3 ± 13.1	32.7 ± 10.3	0.09
LVMi (g/m^2.7^)	40.5 ± 12.5	31.9 ± 4.6	0.12
LV EF (%)	64.3 ± 03.5	63.6 ± 4.5	0.69
GLS (%)	22.3 ± 2.1	21.1 ± 1.5	0.17
Transmitral E/A ratio	1.5 ± 0.21	1.3 ± 0.54	0.14
E velocity DT (msec)	187.3 ± 39.5	194.6 ± 55.5	0.70
E’ velocity (cmsec)	0.70 ± 1.5	0.78 ± 0.8	0.09
E/e’ ratio	5.9 ± 1.1	5.1 ± 0.9	0.08
RWT	0.36 ± 0.6	0.32 ± 0.5	0.23
LAVi (mL/m^2^)	31.4 ± 10.0	26.2 ± 5.2	0.17
PALS	29.4 ± 6.7	37.4 ± 3.9	0.0003
PACS	13.9 ± 2.8	13.5 ± 4.2	0.80

FS = Fazekas score, AFD = Anderson-Fabry disease, LVMi = left ventricular mass index, LV EF = Left ventricular ejection fraction, GLS = Global longitudinal strain, DT= deceleration time, RWT = relative wall thickness, PASP = pulmonary artery systolic pressure, LAVi = Left atrial volume index, PALS = Peak atrial longitudinal strain, PACS = Peak atrial contraction strain.
